# Uptake of Cadmium, Lead and Arsenic by *Tenebrio molitor* and *Hermetia illucens* from Contaminated Substrates

**DOI:** 10.1371/journal.pone.0166186

**Published:** 2016-11-15

**Authors:** H. J. van der Fels-Klerx, L. Camenzuli, M. K. van der Lee, D. G. A. B. Oonincx

**Affiliations:** 1 RIKILT Wageningen University and Research, Akkermaalsbos 2, NL-6708 WB, Wageningen, the Netherlands; 2 Department Entomology, Wageningen University, Wageningen, the Netherlands; The University of Texas at El Paso, UNITED STATES

## Abstract

Insects have potential as a novel source of protein in feed and food production in Europe, provided they can be used safely. To date, limited information is available on the safety of insects, and toxic elements are one of the potential hazards of concern. Therefore, we aimed to investigate the potential accumulation of cadmium, lead and arsenic in larvae of two insect species, *Tenebrio molitor* (yellow mealworm) and *Hermetia illucens* (black soldier fly), which seem to hold potential as a source of food or feed. An experiment was designed with 14 treatments, each in triplicate, per insect species. Twelve treatments used feed that was spiked with cadmium, lead or arsenic at 0.5, 1 and 2 times the respective maximum allowable levels (ML) in complete feed, as established by the European Commission (EC). Two of the 14 treatments consisted of controls, using non-spiked feed. All insects per container (replicate) were harvested when the first larva in that container had completed its larval stage. Development time, survival rates and fresh weights were similar over all treatments, except for development time and total live weight of the half of the maximum limit treatment for cadmium of the black soldier fly. Bioaccumulation (bioaccumulation factor > 1) was seen in all treatments (including two controls) for lead and cadmium in black soldier fly larvae, and for the three arsenic treatments in the yellow mealworm larvae. In the three cadmium treatments, concentrations of cadmium in black soldier fly larvae are higher than the current EC maximum limit for feed materials. The same was seen for the 1.0 and 2.0 ML treatments of arsenic in the yellow mealworm larvae. From this study, it can be concluded that if insects are used as feed materials, the maximum limits of these elements in complete feed should be revised per insect species.

## Introduction

Given the increasing global human population and the increasing consumer demand for (animal derived) proteins, additional (novel) sources of protein, such as insects, are being considered. In Europe, certain insect species are considered for partly replacing conventional sources of animal protein in the food and feed industry. A main advantage of insects over conventional production animals is that they have a high feed conversion efficiency [1]. Furthermore, their feed can be composed of industrial by-products [2–4]. For these reasons, the production of certain insects is more sustainable than conventional sources of protein, considering land use, water use and greenhouse gas emissions [5–8]. Certain insect species, such as the larvae of yellow mealworms (YMW) and black soldier flies (BSF) are a source of high quality protein, as well as certain vitamins and minerals [9–11]. Hence, these insect species are expected to become established as a feed and/or food source on the European market in the future. However, before insects can be used as a (mainstream) source of proteins in Europe, the safety of their use in feed and/or food should be proven. At this moment little information is available on the microbiological and chemical safety of reared insects. Scientific reviews on the safety of using insects for feed and food indicate that accumulation of toxic elements is one of the potential hazards associated with insect production [12–14]. Larvae of four fly species, sourced from producers worldwide, were analysed for the presence of 48 heavy metals and trace elements [15]. Cadmium and arsenic were found to be present–above the limit of detection of the analytical method used—in all samples. In all samples from *Musca domestica*, the cadmium concentration was above the EC maximum limit (ML) for this element in complete animal feed, as specified in 2002/32/EC (EC, 2002). However, the heavy metal and trace element concentrations in the larval feed were not provided. Therefore it was not possible to determine to which extend cadmium and arsenic, and possibly other elements, were accumulated in the larvae. Diener, Zurbrügg [16] investigated the bioaccumulation of cadmium, lead and zinc in BSF, at three different concentrations of these heavy metals. They confirmed that cadmium accumulated in BSF larvae, whereas zinc and lead were excreted. Several studies investigated the potential accumulation of arsenic in insects and indicated that in certain species accumulation occurs [17].

Based on safety assessments of production animals and pets, ML for the presence of several heavy metals and arsenic in feed materials have been established by the European Commission (EC) (2002/32/EC). Because the metabolism of insects may differ from conventional production animals, these ML may not be appropriate for insects. In the study of Diener, Zurbrügg [16], the cadmium concentrations in the feed provided to the larvae were 4, 20 and 100 times the EC maximum limit in complete feed, and 1, 5 and 50 times the EC maximum limit for lead in feed. Although in certain geographical locations these concentrations could occur in feed ingredients, they seem high compared to what would be expected in feed for insects available on the European market. Therefore, the aim of this study was to investigate the accumulation of cadmium, lead and arsenic provided to larvae of the yellow mealworm (*Tenebrio molitor*) and the black soldier fly (*Hermetia illucens*), using feed contaminated at the level of the respective EC maximum limit for complete feed of the particular element, as well as at half and double this limit. The species were chosen because they have been identified as two of the most interesting species for largescale production of feed materials [18]. Hence, we investigated whether the current EC maximum limits in complete feed are suitable for these two insect species.

## Materials and Methods

### Insects

Yellow mealworm (YMW) larvae (*Tenebrio molitor*; Coleoptera: Tenebrionidae) were provided by Kreca V.O.F (Ermelo, The Netherlands). Black soldier fly (BSF) eggs (*Hermetia illucens*; Diptera: Stratiomyidae) were collected from colonies maintained at the Laboratory of Entomology, Wageningen University and Research (Wageningen, The Netherlands).

### Feed spiking

Complete feeds were obtained for both species. For the BSF, chicken feed (Opfokmeel farmfood; Agruniek Rijnvallei Voer B.V., Wageningen, the Netherlands), which has been used for the BSF colony for over six years, was used. For the YMW, a grain mixture for large scale production by Van de Ven Insectenkwekerij (Deurne, The Netherlands) was used. Prior to spiking, the original feed was chemically analysed for the presence of the three elements considered. Spiking was then performed to reach the desired concentration of each element. 100 g of each feed was spiked individually with a 2% nitric acid (HNO_3_) solution containing cadmium, lead or arsenic to concentrations as listed in Table 1. The spiked feed was then thoroughly mixed.

**Table 1:** Concentrations of arsenic, cadmium and lead (mg/kg) in diets provided to larvae of black soldier flies and yellow mealworms, based on the EC maximum level (ML) in complete feed with a moisture content of 12% as defined in Directive 2002/32/EC (EC 2002).

	½ ML	1.0 ML	2.0 ML
Arsenic	1.00	2.00	4.00
Cadmium	0.25	0.50	1.00
Lead	2.50	5.00	10.00

After spiking, homogeneity tests were performed on the feed spiked at 1.0ML (EC maximum limit in complete feed), in order to verify that the feed was spiked homogenously. Ten samples of the spiked feed of each element were analysed according to the method described below.

In addition to the 9 different spiked feeds (3 elements x 3 concentrations), 2 control feeds were prepared. One control consisted of the original feed (control feed), and the other one consisted of the original feed treated with the diluted acid (2% nitric acid solution) (control acid feed).

### Chemical analyses

Feed samples were pre-treated using acid digestion with a microwave oven (MARS express). For the microwave digestion, 0.8g of the sample was mixed with 10ml in concentrated nitric acid (nitric acid (HNO_3_) 69% (m/m) bv. J.T. Baker 9601 “Intra Analysed”) and heated in a microwave oven to a temperature of 210°C. The digests were quantitatively transferred to 50 ml poly propylene (PP) tubes (Greiner Bio-One CENTRIFUGE TUBE 50 ml tube) and diluted with de-ionized water to a final volume of 50 ml. The determination of cadmium, lead and arsenic concentration was done using an Electrothermal atomic absorption spectrophotometer (ETAAS, Aanalyst 800, Perkin Elmer), equipped with a graphite furnace and suitable for element determinations in solid samples [19]. Cadmium, lead and arsenic were measured at wavelengths of 228.8; 283.3 and 193.7 nm respectively. To improve the analytical measurements a 0.1% Pd and 0.12% Mg(NO_3_)_2_ matrix modifier was used. The detection limit (LOD) for the these elements was 0.1 mg/kg.

### Experimental set-up

In total 14 treatments were set up per insect species, each with three replicates. Two control treatments were used. The first control consisted of provision of the original feed (control feed). The second control treatment, named control acid feed, used the original feed treated with diluted acid, but without added elements. The second control was used in order to evaluate the effect of acid addition, used to spike the feed. For the arsenic, cadmium and lead treatments, feed was spiked to concentrations of ½, 1 and twice the EC maximum level (ML), as based on Directive 2002/32/EC (for complete feed with a moisture content of 12%; Table 1). In order to distinguish between elements retained in the feed in the insects’ gut and elements transferred to insect tissue, an extra set of the 2.0 ML treatment was set up for each element. At the end of the experiment, these insects were provided with the original feed (control feed) for two days, allowing them to empty their guts from the contaminated feed. This treatments was called 2.0ML original feed. Thus, the total of 14 treatments included the nine treatments for the 3 elements each at 3 concentrations, three treatments for the 2.0 ML of each element followed by 2 days of control feed (2.0 ML original feed), and two treatments as controls (control feed, control acid feed).

#### Black soldier fly

Each replicate comprised 100 BSF larvae (< 24 hours old) in a plastic container (17.8 x 11.4 x 6.5 cm). The lids were manually perforated to allow sufficient air flow. To each container, 18 g of feed, spiked to the appropriate level, was added. The added feed was mixed with 36ml water (water source).

#### Yellow mealworm

Each replicate comprised 50 YMW larvae (approx. 3 weeks old) in a plastic container (17.9 x 9.3 x 6.3 cm) with aeration slits on the sides allowing air flow. To each container, 8g of feed, spiked to the appropriate level, was added. Also, 0.3 to 0.5g of apple was added as a water source to each container and replaced 3 times per week, allowing *ad libitum* consumption. A total of four apples were used, which were obtained from a single batch and were analysed for the presence of the cadmium, lead and arsenic prior to their use.

Both species were placed in a climate chamber at 27°C, with a relative humidity of 70% and a photoperiod of 12 hours. The climate chamber was illuminated with cool white fluorescent tubes (TLD18W840NG, Philips, Eindhoven, The Netherlands). Depending on the exact position in the tray this resulted in a light intensity in the plastic containers ~7–14 μmol/m2/s (Luxmeter LX1010BS; Uzman Import-export GMBH, Bocholt, Germany). When the first specimen had completed its larval stage in a container, all insects were harvested in accordance with Oonincx et al. (2015). For the 2.0ML original feed treatments, the insects were transferred to a new container, supplied with control diet for two days, and then harvested as above.

For both species, possible differences between the treatments in development time and survival rates of the insects were examined. The development time was defined as the number of days between the start of the experiment and the day the first prepupae were observed. Survival rate was defined as the number of live insect larvae at the end of the experiment divided by this number at the beginning of the experiment.

At harvest, insects were separated from the residual material, consisting of a mixture of excreta and feed. Both insects and the residual material were ground with a batch mill (Ika Labortechnik, Staufen, Germany) and frozen at –20°C. These were subsequently freeze-dried and stored at –80°C until further analyses.

### Analyses of insects

After harvesting, the insects and the residual material were analysed for the respective element concentrations. The BSF were rinsed with tap water to remove surface contamination prior to further analysis. The insect samples were weighed (per container/replicate) and then freeze-dried and re-weighed to determine their dry matter content. Concentrations of the relevant element (cadmium, lead or arsenic) were determined at the Laboratory of RIKILT, Wageningen (The Netherlands) as previously described.

### Data analysis

One way analysis of variance (ANOVA) was used to determine differences between the means of the experimental treatments (means of triplicates) for development time, survival rate, total live weight and dry matter of the larvae, with a significance value of 0.05, using the statistical software package IBM SPPS Statistics 23.

The bioaccumulation factor (BAF), adapted from Walker (1990), was calculated on a dry matter (DM) basis, as BAF = concentration in the organism (DM) /concentration in the feed provided (DM). Thus, a BAF greater than 1 implies bioaccumulation of the element from the substrate into the insect. Statistical differences between the BAF for the different treatments per element were also tested (p<0.05).

## Results

### Feed

Concentrations of Cd, Pb and As in the apples provided to the YMW larvae were below the LOD of the analytical method. Concentrations of Cd, Pb and As in the feed–prior to spiking–were 0.06 mg/kg Cd, 0.15 mg/kg Pb and <0.1 mg/kg As in the BSF feed, and 0.13 mg/kg Cd, <0.1 mg/kg Pb and <0.1 mg/kg As in the mealworm feed. Moisture contents were 14.1% and 11.1%, respectively.

Homogeneity tests showed that for both the BSF and YMW feed the elements were homogenously distributed in the respective 1ML feed batches. The feed materials that were spiked at 0.5ML and 2ML were assumed to be homogenous as well since they were prepared in the same way.

### Development of insects

Table 2 presents the results on the development time, survival, total live weight and dry matter (DM) content of the different treatments. For BSF, the development time was 12–14 days on most treatments, except for the Cd 0.5ML treatment which had a significant longer development time (21 days). Larvae in this treatment also had a significant lower live weight (mean 4.2 +/- 0.37g) compared to all other treatments, which ranged from 9.6–11.9g. These differences in development time and total live weight for the Cd 0.5ML treatment were significant different (p<0.05) with all other treatments. Survival rates of BSF were similar between all treatments, and averaged 83.7 to 97.3%, as was the DM content.

**Table 2:** Survival, development time, total live weight and dry matter percentage for larvae of black soldier fly (*Hermetia illucens*) and yellow mealworm (*Tenebrio molitor*); data presented as mean ± SD; n = 3. Full data are presented in S1 Table. Animals were provided with either a control diet, a control diet containing a vehicle (acid) or a diet spiked with arsenic (As), lead (Pb) or cadmium (Cd) at the maximum level (ML), half the ML or twice the ML for complete feed with a moisture content of 12%, as defined in Directive 2002/32/EC (EC 2002). No superscripts in common within a column indicates significant differences (ANOVA followed by Tukeys HSD posthoc test; P<0.05).

	Black soldier fly				Yellow mealworm			
	Survival	Development time	Total live weight	Dry matter	Survival	Development time	Total live weight	Dry matter
	(%)	(days)	(g)	(% live weight)	(%)	(days)	(g)	(% live weight)
Control	81.3 ± 10.02^a^	12.3 ± 0.58^a^	9.6 ± 0.79^b^	25.7 ± 0.08^a,b^	80.7 ± 8.33^b^	47.0 ± 3.61^a^	4.7 ± 0.89^b^	34.0 ± 1.17^a^
Control acid	93.7 ± 1.15^a^	12.7 ± 0.58^a^	10.3 ± 1.60^b^	24.4 ± 5.17^a,b^	53.3 ± 11.02^a,b^	42.7 ± 9.07^a^	2.1 ± 0.49^a^	33.3 ± 1.40^a^
As 1/2 ML	97.3 ± 3.06^a^	13.3 ± 1.15^a^	11.9 ± 1.16^b^	27.9 ± 1.28^a,b^	48.0 ± 13.86^a,b^	46.3 ± 4.73^a^	2.3 ± 0.46^a^	33.8 ± 0.88^a^
As 1 ML	91.3 ± 8.50^a^	14.0 ± 0.00^a^	11.3 ± 1.45^b^	29.7 ± 1.29^b^	52.0 ± 15.10^a,b^	45.0 ± 3.61^a^	2.3 ± 0.79^a^	34.1 ± 0.88^a^
As 2 ML	91.0 ± 10.6^a^	13.7 ± 0.58^a^	11.0 ± 1.65^b^	26.8 ± 0.83^a,b^	52.0 ± 8.72^a,b^	42.7 ± 6.35^a^	2.0 ± 0.42^a^	33.4 ± 1.19^a^
Pb 1/2 L	90.7 ± 9.45^a^	13.7 ± 0.58^a^	11.1 ± 0.82^b^	29.1 ± 1.15^b^	55.3 ± 11.55^a,b^	46.7 ± 8.02^a^	2.7 ± 0.38^a^	34.2 ± 0.76^a^
Pb 1 ML	94.7 ± 6.43^a^	13.7 ± 0.58^a^	10.8 ± 1.50^b^	28.5 ± 0.59^a,b^	46.7 ± 16.04^a,b^	47.3 ± 9.81^a^	2.4 ± 0.61^a^	34.4 ± 0.69^a^
Pb 2 ML	94.7 ± 4.04^a^	13.7 ± 0.58^a^	11.1 ± 0.75^b^	27.1 ± 2.01^a,b^	66.7 ± 23.86^a,b^	47.7 ± 8.50^a^	3.4 ± 0.89^a,b^	34.5 ± 1.20^a^
Cd 1/2 ML	83.7 ± 10.21^a^	21.0 ± 0.00^b^	4.2 ± 0.37^a^	20.7 ± 5.06^a^	41.3 ± 3.06^a^	45.0 ± 7.21^a^	2.0 ± 0.18^a^	33.3 ± 0.42^a^
Cd 1 ML	93.3 ± 5.03^a^	12.3 ± 0.58^a^	10.5 ± 0.57^b^	27.9 ± 4.18^a,b^	68.7 ± 9.24^a,b^	45.0 ± 3.61^a^	3.6 ± 0.22^a,b^	33.9 ± 0.24^a^
Cd 2 ML	91.7 ± 7.09^a^	13.3 ± 0.58^a^	10.5 ± 1.11^b^	25.3 ± 2.22^a,b^	64.0 ± 8.72^a,b^	45.7 ± 4.51^a^	3.3 ± 0.97^a,b^	34.2 ± 0.73^a^

For YMW, the development time was unaffected by treatment (p<0.05), with averages ranging from 45.0–47.7 days, as was the DM content (33.3–34.1%). The total live weight of the YMW larvae ranged from 2.0g (Cd 0.5 ML) to -4.7 (control). The average survival rate was 59%, ranging from 41.3% (Cd 0.5ML) to 80.7% (control). The survival rate was significantly lower for the Cd 0.5ML treatment, as compared to the control (p<0.05).

### Element concentrations

Based on colour and structure differences between feed and excreta, the residual material after harvest primarily consisted of insect excreta, as opposed to feed left overs.

**Fig 1:**
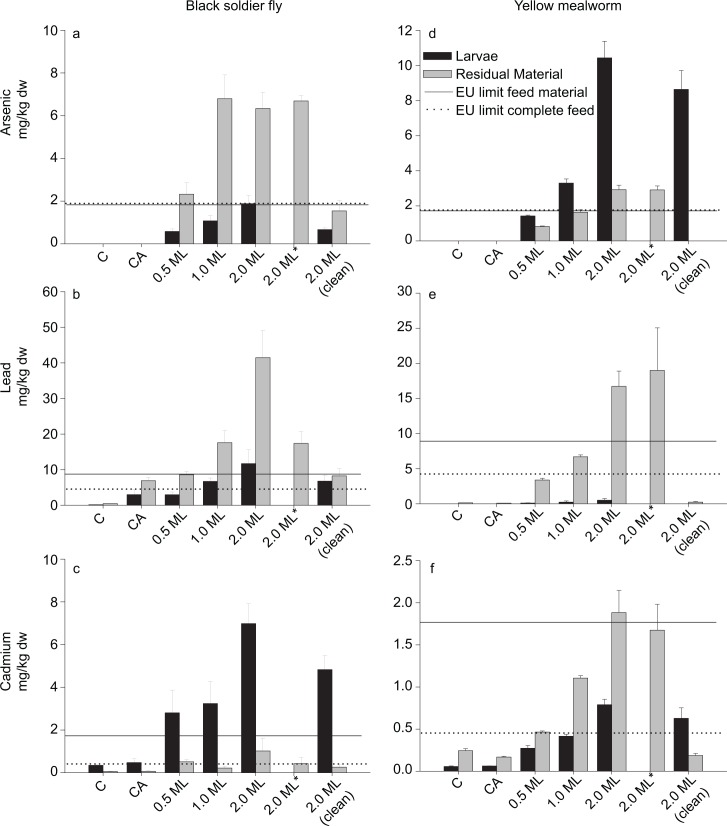
Concentrations of As, Pb and Cd in larvae (black bars) and residual material (grey bars) of the black soldier fly and the yellow mealworm (mean ± sd of three replicates). Provided with control feed (C), control feed with acid (CA), or feed containing 0.5,1.0 or 2.0 the EC maximum limit (ML). 2.0ML*: concentration in the residual material after transferring larvae to a clean container. 2.0ML (clean): concentrations after 2 days with original (clean) feed. The EC concentration limits of arsenic, lead and cadmium in feed materials (—) and in complete feed (…) (Directive 2002/32/EC). Full data are presented in S1 Table.

Concentrations of Pb and As in BSF residual material were higher than in the BSF larvae themselves, indicating that these two elements were not retained by this species (Fig 1A and 1B). Provision of higher concentrations of these elements resulted in higher concentrations in both the BSF larvae and the residual material. Concentrations of Pb and As in the BSF in the 2.0ML original feed treatment were lower than in the corresponding 2.0ML treatment indicating that at least part of these elements found in the BSF larvae was due to the presence of these elements in their gut load. Conversely, concentrations of Cd in the BSF larvae were consistently higher than in the corresponding residual material, including the 2.0ML original feed treatment, suggesting that this element was incorporated in the BSF body (Fig 1C).

For the YMW, As concentrations were lower in the residual material than in the larvae indicating that this species retains As (Fig 1D). In the 2.0ML original feed treatment, the concentration of As in the residual material was below the LOD, whereas the concentration in the YMW was similar to those in the 2.0ML treatment, indicating that As was incorporated in the YMW body. The reverse is true for Pb and Cd (Fig 1E and 1F). This is especially visible for Pb, where concentrations in the YMW residual material was up to sixty times the concentration found in in the corresponding YMW.

BSF larvae provided with the 2.0ML Pb and As treatment feeds had concentrations above or around the EC limit for feed materials for these two elements. However after being provided with original feed for two days, these concentrations were below the threshold (Fig 1A and 1B). When BSF were provided with Cd contaminated feed at 0.5 ML, their concentrations were above the EC limit for feed material (Fig 1C). With treatments at 1.0 and 2.0 ML this tendency was even more clear. Provision of an original substrate (2.0ML original feed) decreased the Cd concentration in the larvae by 30%. BSF development on the 0.5ML Cd treatment was prolonged and average body weight decreased, whereas this was not the case for the higher concentrations. These inconsistent results coincided with differences in colour, smell and texture of the residual material, indicating that other, unknown factors might have influenced these results.

YMW in the As 0.5ML treatment had concentrations below the EC limit, whereas in the 1.0ML and the 2.0ML concentrations were above this EC limit. Conversely, all Pb and Cd treatments resulted in concentrations in the larvae below the EC limit for feed materials.

### Bioaccumulation factors

Table 3 presents the mean BAF and standard deviation, for both insect species. The BAF varied by insect and by element. For Pb, the BAF was not significantly different (p<0.05) between the treatments of each of BSF and YMW. It was larger than 1 for all BSF treatments—indicating bio-accumulation of this element from the feed into this species—and smaller than 1 for the three Pb treatments of YMW. For Cd, the BAF were significantly larger in the three Cd treatments as compared to the two controls of the YMW, but all were lower than 1. For the BSF, no differences were seen between the three Cd treatments and their controls, and in all cases, the BAF was larger than 1. For As, the BAF of the 2.0 ML treatment of YMW was significantly higher than the BAF of the two other As treatments of this species. The BAF of all three As treatments of YMW were greater than 1 whereas the BAF of the three As treatments of the BSF were smaller than 1.

**Table 3:** Bioaccumulation factor (BAF) for BSF and YMW for five treatments calculated on a dry weight basis (n/a = not applicable due to concentrations below the limit of detection). No superscripts in common within a column indicates significant differences (ANOVA followed by Turkeys HSD posthoc text, with p <0.05).

	Black soldier fly			Mealworm		
	As	Pb	Cd	As	Pb	Cd
Control	n/a	1.1 ± 0.05^a^	5.8 ± 1.0^a^	n/a	n/a	0.43 ± 0.039^a^
Control acid	n/a	1.8 ± 0.81^a^	8.1 ± 2.9^a^	n/a	n/a	0.48 ± 0.009^a^
0.5 ML	0.58 ± 0.12^a^	1.2 ± 0.30^a^	9.5 ± 3.6^a^	1.4 ± 0.045^a^	0.043 ± 0.013^a^	0.71 ± 0.083^b^
1.0 ML	0.56 ± 0.13^a^	1.4 ± 0.20^a^	6.1 ± 1.9^a^	1.6 ± 0.11^a^	0.046 ± 0.032^a^	0.65 ± 0.037^b^
2.0 ML	0.49 ± 0.10^a^	1.2 ± 0.40^a^	6.9 ± 0.92^a^	2.6 ± 0.23^b^	0.051 ± 0.022^a^	0.69 ± 0.056^b^

## Discussion and Conclusion

The development time of insects depends on several factors, such as species, temperature, diet, and population density [20–22]. The development time and live weight of the BSF were similar to these parameters in the study by _Diener, Zurbrügg [23]. Also in that study, the larvae fed on chicken feed. Hence, the housing and feeding regimes employed in this study were appropriate for both the BSF and YMW. Acid addition did not affect development time or survival rates as compared to the control. Total live larval weights, development time and survival time did not differ between the 14 treatments of each of BSF and YMW, except for the development time and total live weight of the Cd 0.5ML treatment of the BSF. BSF development on this Cd treatment was prolonged and average body weight decreased, whereas this was not the case for the higher concentrations. These inconsistent results coincided with differences in colour, smell and texture of the residual material, indicating that other, unknown factors might have influenced these results.

Different accumulation patterns of As and the two heavy metals, Cd and Pb, were observed for the BSF and YMW. BSF showed highest accumulation of Cd, followed by Pb. In YMW, accumulation of As was highest, and low accumulation was seen for Pb.

Bioaccumulation was significantly different between the particular element and their control, only for the three Cd treatments and the 2.0ML As treatment of YMW. Element uptake does not seem to be strongly regulated in insects [24]. After uptake, the element can be bound to metallothioneins or sequestered in vesicles, which effectively inactivates these metals. In certain cases these are excreted by means of exocytose into the lumen of the digestive tract [24, 25].

**Cadmium** accumulated in the BSF, such that in every treatment, the concentration in the larvae was higher than in the residual material, which concurs with the findings of _Diener, Zurbrügg [16]. This pattern of Cd accumulation was less apparent in the YMW. Previous studies have found large differences in the ability to excrete Cd between species. In a previous study on two species of Hymenoptera and three species of Lepidoptera, effective excretion of Cd was observed [26], whereas in a species of Homopteran, Cd accumulated in the insect [27]. Migratory locusts (Orthopera) are able to keep stable Cd body concentrations for a short period, after which Cd is accumulated [28]. In Coleoptera, increased dietary Cd levels lead to increased Cd body concentrations [29–31], as was observed in the YMW in this study (significant difference between Cd treatments and controls). In the YMW two pools of Cd exist; a small proportion penetrates the epithelium through Ca^2+^ channels to reach other tissues [30, 32], while most of the Cd is stored in the gut epithelium and bound by a cadmium-binding protein [33]. The cells of the midgut epithelium in this species have a four day lifespan, after which their contents, including the bound Cd, is released into the lumen of the gut and, subsequently, excreted in the faeces [30, 33]. However, the fraction of Cd that has penetrated the gut and is distributed amongst other organs is retained longer [30, 33]. This concurs with our results, as Cd did not bioaccumulate (BAF < 1) in the YMW, and after being provided with original feed, Cd was excreted.

In our study, Cd accumulated in the BSF (BAF > 1), and in fact, Cd accumulates within various Dipteran species [16, 34–39]. Both in fruit flies and in marsh mosquitoes, high dietary Cd concentrations increased metallothionein levels, which effectively bind to the Cd resulting in an increased storage capacity [36, 39]. In most Diptera, this increased capacity does not lead to increased Cd excretion. However, there are natural strains of midges and fruit flies which have a duplication of the metallothionein gene that does increase excretion efficiency, and leads to a subsequent higher resistance to dietary Cd [25, 40]. Additionally, studies suggest that Cd can be transported by means of heat shock proteins, and that Cd is able to pass through Ca^2+^ channels [32, 41]. Because larvae of the BSF have an exceptionally high Ca content compared to other insect species, they might accumulate Cd more strongly than other Dipterans [10]. Moreover, differences between Diptera and Coleoptera regarding Cd transport and storage probably explain the difference in Cd accumulation between the YMW and BSF, as reflected by their BAF (<0.7 versus >6, respectively). A study in which Cd contaminated material was provided to Dipteran larvae, which in turn were provided as feed to a predatory Coleopteran, reported Cd accumulation in the Dipteran, but lower Cd concentrations in the Coleopteran, further supporting the concept that Coleoptera can be classified as Cd deconcentrators and Diptera classified as Cd macroconcentrators [24, 38].

**Lead** concentrations in the BSF were similar to the concentration in the feed (BAF ~ 1.2–1.4 for the three Pb treatments), but the BAF was in the same range in the controls. _Diener, Zurbrügg [16] reported higher Pb concentrations in the larval exuviae than in the larvae or their feed, indicating that Pb is sequestered in the exoskeleton of the BSF. In the YMW, the Pb concentration was far lower than in their feed (BAF ~0.05). It appears that the storage site in YMW also differs from the BSF, because Pb concentrations in larval exuviae are lower than in the larval body [31]. In the burrowing mayfly, an aquatic Ephemeropteran, a large proportion of the Pb was found on the body surface and not in the gut, which might indicate poor absorption of Pb in that species [42]. In the Orthopteran *Aiolopus thalassinus*, Pb concentrations were four times higher in the wings than in the gut [43]. Whether this Pb was incorporated in the wings, or attached to the surface is not clear.

**Arsenic** was detected in the BSF larvae of the three As treatments, however, the majority of consumed arsenic was excreted (BAF ~0.5–0.6). Excretion continued after the larvae were given original feed, such that after two days As concentrations in the larvae decreased by 65%, indicating that the arsenic was present inside the gut. Conversely, the YMW accumulated As, leading to increasing concentrations in the insect with increasing feed concentrations, but also an increasing BAF (1.4–2.6). This could indicate that YMW can excrete As, but that this mechanism has a limited capacity. With this species, provision of original feed for two days resulted in a concentration reduction of 17%, indicating that the majority of As was accumulated within the YMW tissue. Whether As is stored within a cell, or by extracellular coagulation is unknown [44]. From field studies it seems that invertebrate As levels depend more strongly on taxonomical differences than on exposure levels [17]. In the latter study Diptera and Orthoptera contained far lower levels than Odonata and Lepidoptera from the same location.

Another study, which determined As in the Mountain Pine beetle (Coleoptera), found approximately double the concentrations in the beetle and its larvae, compared to As concentrations in the phloem of its host plant [45]. Gongalsky, Chudnyavtseva [46] compared As concentrations in four Coleopterans from either As polluted areas or an unpolluted control site. They found elevated As concentrations in all beetle species from the polluted areas. The tenebrionid in that study had a 10-fold As concentration compared to the other species, which could be due to dietary differences. The difference in As accumulation and excretion found in this study for YMW and BSF seems consistent with patterns described for Coleoptera and Diptera in other studies.

Because BSF and YMW are expected to enter the European food and feed market, it is important to compare the relation between the ML in complete feed with the ML in feed materials for heavy metals and arsenic with respect to these insect species (Directive 2002/32/EC). The results of this study show clear differences between species and elements. For instance, the EC limit for As in complete feed (2 mg kg^-1^) is appropriate for the BSF, but YMW provided with feed at this limit surpass the EC limit for feed materials. On the other hand, the EC limit for Cd in complete feed is appropriate for YMW, whereas BSF fed at this maximum limit for Cd, result in a concentration exceeding the EC limit for Cd in feed materials. Conversely, the current EC maximum limit for Pb in complete feed resulted in BSF and YMW larvae below the ML for feed materials. Hence for lead, the current EC limits seem to be safe for both species. Given the different responses to heavy metals and arsenic in feed of these two insect species, the ML of these elements in complete feed need to be re-established, per insect order, or possibly insect species, when they are reared for use as feed materials.

## Supporting Information

S1 Table Raw data for Supporting Information. (PDF)

## References

[1] Rumpold BA, Schlüter OK. Potential and challenges of insects as an innovative source for food and feed production. Innovative Food Science & Emerging Technologies. 2013;17:1–11.

[2] Van Huis A, Van Itterbeeck J, Klunder H, Mertens E, Halloran A, Muir G, et al Edible insects: future prospects for food and feed security: Food and agriculture organization of the United nations (FAO); 2013.

[3] Oonincx DGAB, van Broekhoven S, van Huis A, van Loon JJA. Feed conversion, survival and development, and composition of four insect species on diets composed of food by-products. PLoS ONE. 2015;10(12):e0144601 10.1371/journal.pone.0144601 26699129 PMC4689427

[4] van Broekhoven S, Oonincx DGAB, van Huis A, van Loon JJA. Growth performance and feed conversion efficiency of three edible mealworm species (Coleoptera: Tenebrionidae) on diets composed of organic by-products. Journal of Insect Physiology. 2015;73(0):1–10. 10.1016/j.jinsphys.2014.12.005 25576652

[5] Miglietta PP, De Leo F, Ruberti M, Massari S. Mealworms for food: A water footprint perspective. Water. 2015;7(11):6190–203.

[6] Oonincx DGAB, de Boer IJM. Environmental impact of the production of mealworms as a protein source for humans–A life cycle assessment. PLoS ONE. 2012;7(12):e51145 10.1371/journal.pone.0051145 23284661 PMC3526541

[7] Oonincx DGAB, van Itterbeeck J, Heetkamp MJ, van den Brand H, van Loon JJ, van Huis A. An exploration on greenhouse gas and ammonia production by insect species suitable for animal or human consumption. PLoS ONE. 2010;5(12):e14445 Epub 2011/01/06. 10.1371/journal.pone.0014445 21206900 PMC3012052

[8] van Zanten HH, Mollenhorst H, Oonincx DG, Bikker P, Meerburg BG, de Boer IJ. From environmental nuisance to environmental opportunity: housefly larvae convert waste to livestock feed. Journal of Cleaner Production. 2015;102(1):362–9.

[9] Rumpold BA, Schlüter OK. Nutrient composition of insects and their potential application in food and feed in Europe. Food Chain. 2014;4(2):129–39.

[10] Finke MD, Oonincx DGAB. Insects as food for insectivores In: Morales-Ramos JA, Rojas MG, Shapiro-Ilan DI, editors. Mass Production of Beneficial Organisms: Invertebrates and Entomopathogens. London, UK: Academic Press; 2013.

[11] Bosch G, Zhang S, Oonincx DGAB, Hendriks WH. Protein quality of insects as potential ingredients for dog and cat foods. Journal of Nutritional Science. 2014;3(e29):1–4. 10.1017/jns.2014.23 26101598 PMC4473158

[12] Belluco S, Losasso C, Maggioletti M, Alonzi CC, Paoletti MG, Ricci A. Edible insects in a food safety and nutritional perspective: A critical review. Comprehensive Reviews in Food Science and Food Safety. 2013;12(3):296–313.

[13] Rumpold BA, Schlüter OK. Nutritional composition and safety aspects of edible insects. Mol Nutr Food Res. 2013;57:802–23. 10.1002/mnfr.201200735 23471778

[14] van der Spiegel M, Noordam MY, van der Fels-Klerx HJ. Safety of novel protein sources (insects, microalgae, seaweed, duckweed, and rapeseed) and legislative aspects for their application in food and feed production. Comprehensive Reviews in Food Science and Food Safety. 2013;12(6):662–78. 10.1111/1541-4337.12032 33412718

[15] Charlton AJ, Dickinson M, Wakefield ME, Fitches E, Kenis M, Han R, et al Exploring the chemical safety of fly larvae as a source of protein for animal feed. Journal of Insects as Food and Feed. 2015;1(1):7–16. 10.3920/jiff2014.0020

[16] Diener S, Zurbrügg C, Tockner K. Bioaccumulation of heavy metals in the black soldier fly, *Hermetia illucens* and effects on its life cycle. Journal of Insects as Food and Feed. 2015;1(4):261–70.

[17] Moriarty MM, Koch I, Gordon RA, Reimer KJ. Arsenic speciation of terrestrial invertebrates. Environmental Science & Technology. 2009;43(13):4818–23. 10.1021/es900086r 19673270

[18] Veldkamp T. Insects as a sustainable feed ingredient in pig and poultry diets: a feasibility study: Wageningen UR Livestock Research; 2012.

[19] Devkota B, Schmidt GH, editors. Bioaccumulation of heavy metals (Hg, Cd, Pb) in different organs of the grasshopper, Aiolopus thalassinus (Fabr.)(Acrididae, Orthoptera). Proceedings of the VIth International Conference on Bioindicatores Deteriorisationes Regionis; 1992: Institute of Landscape Ecology CAS Ceśke Budéjovice.

[20] Weaver DK, McFarlane JE. The effect of larval density on growth and development of *Tenebrio molitor*. Journal of Insect Physiology. 1990;36(7):531–6. 10.1016/0022-1910(90)90105-O

[21] Tomberlin JK, Adler PH, Myers HM. Development of the black soldier fly (Diptera: Stratiomyidae) in relation to temperature. Environmental Entomology. 2009;38(3):930–4. 10.1603/022.038.0347 19508804

[22] Loudon C. Development of *Tenebrio molitor* in low oxygen levels. Journal of Insect Physiology. 1988;34(2):97–103. 10.1016/0022-1910(88)90160-6

[23] Diener S, Zurbrügg C, Tockner K. Conversion of organic material by black soldier fly larvae: Establishing optimal feeding rates. Waste Management & Research. 2009;27(6):603–10. 10.1177/0734242x09103838 19502252

[24] Dallinger R. Strategies of metal detoxification in terrestrial invertebrates. Ecotoxicology of Metals in Invertebrates. 1993;245.

[25] Postma JF, van Nugteren P, de Jong MBB. Increased cadmium excretion in metal‐adapted populations of the midge *Chironomus riparius* (diptera). Environmental Toxicology and Chemistry. 1996;15(3):332–9.

[26] Lindqvist L. Accumulation of cadmium, copper, and zinc in five species of phytophagous insects. Environmental Entomology. 1992;21(1):160–3. 10.1093/ee/21.1.160

[27] Crawford L, Hodkinson I, Lepp N. The effects of elevated host-plant cadmium and copper on the performance of the aphid *Aphis fabae* (Homoptera: Aphididae). Journal of Applied Ecology. 1995:528–35.

[28] Crawford LA, Lepp NW, Hodkinson ID. Accumulation and egestion of dietary copper and cadmium by the grasshopper *Locusta migratoria* R & F (Orthoptera: Acrididae). Environmental Pollution. 1996;92(3):241–6. 15091374 10.1016/0269-7491(96)00004-8

[29] Hunter B, Johnson M, Thompson D. Ecotoxicology of copper and cadmium in a contaminated grassland ecosystem. II. Invertebrates. Journal of Applied Ecology. 1987:587–99.

[30] Lindqvist L, Block M. Excretion of cadmium during moulting and metamorphosis in *Tenebrio molitor* (Coleoptera; Tenebrionidae). Comparative Biochemistry and Physiology Part C: Pharmacology, Toxicology and Endocrinology. 1995;111(2):325–8. 10.1016/0742-8413(95)00057-U

[31] Vijver M, Jager T, Posthuma L, Peijnenburg W. Metal uptake from soils and soil–sediment mixtures by larvae of *Tenebrio molitor* (L.) (Coleoptera). Ecotoxicology and Environmental Safety. 2003;54(3):277–89. 10.1016/S0147-6513(02)00027-1 12651183

[32] Braeckman B, Smagghe G, Brutsaert N, Cornelis R, Raes H. Cadmium uptake and defense mechanism in insect cells. Environmental Research. 1999;80(3):231–43. 10.1006/enrs.1998.3897 10092443

[33] Pedersen SA, Kristiansen E, Andersen RA, Zachariassen KE. Cadmium is deposited in the gut content of larvae of the beetle *Tenebrio molitor* and involves a Cd-binding protein of the low cysteine type. Comparative Biochemistry and Physiology Part C: Toxicology & Pharmacology. 2008;148(3):217–22. 10.1016/j.cbpc.2008.05.013 18603479

[34] Wu GX, Ye GY, Hu C, Cheng JA. Accumulation of cadmium and its effects on growth, development and hemolymph biochemical compositions in *Boettcherisca peregrina* larvae (Diptera: Sarcophagidae). Insect Science. 2006;13(1):31–9.

[35] Yasunobu A, Suzuki KT. Excretion of cadmium and change in the relative ratio of iso-cadmium-binding proteins during metamorphosis of fleshfly (*Sarcophaga peregrina*). Comparative Biochemistry and Physiology Part C: Comparative Pharmacology. 1984;78(2):315–7. 10.1016/0742-8413(84)90089-6 6149071

[36] Maroni G, Watson D. Uptake and binding of cadmium, copper and zinc by *Drosophila melanogaster* larvae. Insect Biochemistry. 1985;15(1):55–63. 10.1016/0020-1790(85)90044-7

[37] Kazimírová M, Ortel J. Metal accumulation by *Ceratitis capitata* (Diptera) and transfer to the parasitic wasp *Coptera occidentalis* (Hymenoptera). Environmental Toxicology and Chemistry. 2000;19(7):1822–9.

[38] Maryanski M, Kramarz P, Laskowski R, Niklinska M. Decreased energetic reserves, morphological changes and accumulation of metals in carabid beetles (*Poecilus cupreus* L.) exposed to zinc-or cadmium-contaminated food. Ecotoxicology. 2002;11(2):127–39. 11990769 10.1023/a:1014425113481

[39] Mireji PO, Keating J, Hassanali A, Impoinvil DE, Mbogo CM, Muturi MN, et al Expression of metallothionein and alpha-tubulin in heavy metal-tolerant *Anopheles gambiae sensu stricto* (Diptera: Culicidae). Ecotoxicolgy and Environmental Safety. 2010;73(1):46–50. Epub 2009/09/09. 10.1016/j.ecoenv.2009.08.004 19735939 PMC2783303

[40] Lauverjat S, Ballan-Dufrancais C, Wegnez M. Detoxification of cadmium Ultrastructural study and electron-probe microanalysis of the midgut in a cadmium-resistant strain of *Drosophila melanogaster*. Biology of Metals. 1989;2(2):97–107. 2518373 10.1007/BF01129208

[41] Craig A, Hare L, Tessier A. Experimental evidence for cadmium uptake via calcium channels in the aquatic insect Chironomus staegeri. Aquatic Toxicology. 1999;44(4):255–62.

[42] Hare L, Saouter E, Campbell PGC, Tessier A, Ribeyre F, Boudou A. Dynamics of cadmium, lead, and zinc exchange between nymphs of the burrowing mayfly *Hexagenia rigida* (Ephemeroptera) and the environment. Canadian Journal of Fisheries and Aquatic Sciences. 1991;48(1):39–47. 10.1139/f91-006

[43] Schmidt GH, Ibrahim NMM. Heavy metal content (Hg2+, Cd2+, Pb2+) in various body parts: Its impact on cholinesterase activity and binding glycoproteins in the grasshopper *Aiolopus thalassinus* adults. Ecotoxicology and Environmental Safety. 1994;29(2):148–64. 10.1016/0147-6513(94)90016-7 7533707

[44] Rahman MM, Rahman F, Sansom L, Naidu R, Schmidt O. Arsenic interactions with lipid particles containing iron. Environmental Geochemistry and Health. 2009;31(1):201–6. 19093213 10.1007/s10653-008-9236-z

[45] Morrissey CA, Albert CA, Dods PL, Cullen WR, Lai VW-M, Elliott JE. Arsenic accumulation in bark beetles and forest birds occupying mountain pine beetle infested stands treated with monosodium methanearsonate. Environmental Science & Technology. 2007;41(4):1494–500. 17593762 10.1021/es061967r

[46] Gongalsky BK, Chudnyavtseva II, Pokarzhevskii DA, Samonov EA, Slobodyan YV. Arsenic bioaccumulation by beetles in an Arsenic-rich region. Bulletin of Environmental Contamination and Toxicology. 2004;72(6):1115–21. 10.1007/s00128-004-0359-3 15362438

