# Supplementary material for: Uptake of Cadmium, Lead and Arsenic by Tenebrio molitor and Hermetia illucens from Contaminated Substrates
Source: PLoS One. 2016 Nov 15;11(11):e0166186. doi: 10.1371/journal.pone.0166186 (PMC5112862; doi:10.1371/journal.pone.0166186)
Supplement: S1 Table — (PDF) [file pone.0166186.s001.pdf]

S1 Table: raw data of experiment as Supporting Information

YMW

Treatment	Larvae		Faeces		Clean faeces		Feed	
	conc (mg/kg_{dw})	%DM	conc (mg/kg_{dw})	%DM	conc (mg/kg_{dw})	%DM	conc (mg/kg_{dw})	%DM
**Control As**	<0.05	33%	<0.05	89%			<0.05	89%
	<0.05	34%	<0.05	89%			<0.05	90%
	<0.05	35%	<0.05	90%			<0.05	90%
**Control Acid As**	<0.05	32%	<0.05	90%			<0.05	93%
	<0.05	35%	<0.05	89%			<0.05	93%
	<0.05	33%	<0.05	91%			<0.05	93%
**As ½ML**	1.47	33%	0.84	89%			1.06	93%
	1.44	34%	0.80	89%			1.06	93%
	1.37	34%	0.85	90%			1.06	93%
**As 1ML**	3.41	35%	1.71	90%			2.06	97%
	3.03	34%	1.47	89%			2.06	97%
	3.47	33%	1.72	90%			2.06	97%
**As 2ML**	9.37	35%	2.65	88%			4.08	94%
	11.16	33%	3.13	90%			4.08	94%
	10.78	33%	3.01	90%			4.08	94%
**As 2ML***	9.86	35%	3.03	90%	<0.05	89%	4.08	94%
	7.79	34%	2.64	90%	<0.05	89%	4.08	94%
	8.27	36%	3.05	90%	<0.05	89%	4.08	94%
**Control Pb**	<0.05	33%	0.15	89%			<0.05	89%
	<0.05	34%	0.13	89%			<0.05	90%
	<0.05	35%	0.13	90%			<0.05	90%
**Control Acid Pb**	<0.05	32%	0.08	90%			<0.05	93%
	<0.05	35%	0.09	89%			<0.05	93%
	<0.05	33%	0.09	91%			<0.05	93%
**Pb ½ML**	0.14	35%	3.36	89%			2.57	94%
	0.11	34%	3.14	90%			2.57	94%
	0.08	34%	3.64	90%			2.57	94%
**Pb 1ML**	0.16	35%	6.45	89%			5.10	93%
	0.12	34%	6.97	90%			5.10	93%
	0.42	34%	6.67	90%			5.10	93%
**Pb 2ML**	0.35	34%	16.31	89%			10.13	94%
	0.41	33%	19.09	90%			10.13	94%
	0.77	36%	14.80	89%			10.13	94%
**Pb 2ML***	<0.05	36%	16.62	90%	0.23	90%	10.13	94%
	<0.05	35%	14.47	90%	0.15	89%	10.13	94%
	<0.05	37%	25.91	90%	0.31	89%	10.13	94%
**Control Cd**	0.05	33%	0.26	89%			0.13	89%
	0.06	34%	0.21	89%			0.13	90%
	0.05	35%	0.26	90%			0.13	90%
**Control Acid Cd**	0.06	32%	0.16	90%			0.13	93%
	0.06	35%	0.18	89%			0.13	93%
	0.06	33%	0.17	91%			0.13	93%
**Cd ½ML**	0.30	33%	0.46	89%			0.38	94%
	0.29	33%	0.48	90%			0.38	94%
	0.24	34%	0.46	90%			0.38	94%
**Cd 1ML**	0.44	34%	1.13	89%			0.63	96%
	0.41	34%	1.11	89%			0.63	96%
	0.40	34%	1.08	89%			0.63	96%
**Cd 2ML**	0.75	34%	1.85	89%			1.14	93%
	0.75	35%	2.16	89%			1.14	93%
	0.86	34%	1.64	90%			1.14	93%
**Cd 2ML***	0.77	35%	1.75	90%	0.18	89%	1.14	93%
	0.59	36%	1.34	90%	0.17	89%	1.14	93%
	0.53	36%	1.94	91%	0.21	89%	1.14	93%

BSF								
Treatment	Larvae		Faeces		Clean faeces		Feed	
	conc (mg/kg_{dw})	%DM	conc (mg/kg_{dw})	%DM	conc (mg/kg_{dw})	%DM	conc (mg/kg_{dw})	%DM
**Control As**	<0.1	25.8%	<0.1	15%			<0.1	85.9%
	<0.1	26%	<0.1	21%			<0.1	85.9%
	<0.1	26%	<0.1	19%			<0.1	85.9%
**Control Acid As**	<0.1	18%	<0.1	18%			<0.1	85.9%
	<0.1	27%	<0.1	22%			<0.1	85.9%
	<0.1	28%	<0.1	20%			<0.1	85.9%
**As ½ML**	0.71	26%	2.29	19%			1.0	85.9%
	0.48	28%	2.88	19%			1.0	85.9%
	0.56	29%	1.80	20%			1.0	85.9%
**As 1ML**	0.82	31%	7.71	17%			1.9	85.9%
	1.11	28%	7.12	19%			1.9	85.9%
	1.31	30%	5.56	18%			1.9	85.9%
**As 2ML**	1.75	26%	5.90	18%			3.8	85.9%
	1.59	28%	5.90	18%			3.8	85.9%
	2.33	26%	7.22	20%			3.8	85.9%
**As 2ML***	0.59	29%	6.89	21%	1.63	19%	3.8	85.9%
	0.70	30%	6.39	20%	2.00	16%	3.8	85.9%
	0.73	29%	6.79	18%	1.01	24%	3.8	85.9%
**Control Pb**	0.15	26%	0.52	15%			0.1	85.9%
	0.17	26%	0.44	21%			0.1	85.9%
	0.16	26%	0.42	19%			0.1	85.9%
**Control Acid Pb**	4.59	18%	7.81	18%			1.7	85.9%
	2.39	27%	6.84	22%			1.7	85.9%
	2.01	28%	6.02	20%			1.7	85.9%
**Pb ½ML**	3.72	28%	9.58	16%			2.5	85.9%
	2.99	29%	8.50	20%			2.5	85.9%
	2.19	30%	7.57	20%			2.5	85.9%
**Pb 1ML**	5.60	29%	21.52	16%			4.9	85.9%
	7.16	29%	15.72	18%			4.9	85.9%
	7.38	28%	15.48	18%			4.9	85.9%
**Pb 2ML**	15.19	25%	33.35	17%			9.6	85.9%
	7.51	29%	48.41	19%			9.6	85.9%
	12.41	28%	42.74	16%			9.6	85.9%
**Pb 2ML***	8.70	28%	20.65	28%	6.20	27%	9.6	85.9%
	5.95	32%	14.07	23%	8.26	32%	9.6	85.9%
	5.75	29%	17.46	18%	10.26	20%	9.6	85.9%
**Control Cd**	0.36	26%	0.08	15%			0.1	85.9%
	0.39	26%	0.04	21%			0.1	85.9%
	0.27	26%	0.06	19%			0.1	85.9%
**Control Acid Cd**	0.67	18%	0.11	18%			0.1	85.9%
	0.40	27%	0.05	22%			0.1	85.9%
	0.35	28%	0.04	20%			0.1	85.9%
**Cd ½ML**	4.02	15%	0.46	32%			0.3	85.9%
	2.06	22%	0.64	22%			0.3	85.9%
	2.33	25%	0.42	30%			0.3	85.9%
**Cd 1ML**	4.05	25%	0.18	19%			0.5	85.9%
	2.08	33%	0.18	19%			0.5	85.9%
	3.60	27%	0.30	17%			0.5	85.9%
**Cd 2ML**	7.37	24%	1.66	18%			1.0	85.9%
	7.65	24%	0.94	17%			1.0	85.9%
	5.92	28%	0.46	17%			1.0	85.9%
**Cd 2ML***	5.39	30%	0.26	24%	0.36	24%	1.0	85.9%
	4.11	29%	0.77	20%	0.09	25%	1.0	85.9%

	4.98	31%	0.24	21%	0.33	18%	1.0	85.9%

Treatment	**BSF**			**YMW**		
	Survival (%)	Development time (days)	Total live weight (g)	Survival (%)	Development time (days)	Total live weight (g)
**Control As**	89%	12	8.79	90%	50	5.63
	85%	12	9.73	74%	43	3.85
	70%	13	10.36	78%	48	4.76
**Control Acid As**	93%	12	8.67	54%	36	1.60
	93%	13	10.34	42%	53	2.57
	95%	13	11.87	64%	39	2.27
**As ½ML**	94%	12	10.53	40%	48	2.06
	98%	14	12.46	40%	50	2.01
	100%	14	12.62	64%	41	2.83
**As 1ML**	100%	14	9.67	38%	46	1.94
	83%	14	12.51	68%	48	3.15
	91%	14	11.59	50%	41	1.66
**As 2ML**	95%	13	9.13	42%	50	2.52
	99%	14	11.39	56%	39	1.71
	79%	14	12.35	58%	39	1.92
**As 2ML***	86%	13	10.45	60%	43	2.49
	106%	13	14.25	66%	43	2.99
	80%	13	13.86	58%	43	3.07
**Pb ½ML**	94%	13	10.19	42%	55	2.69
	98%	14	11.68	62%	46	2.38
	80%	14	11.53	62%	39	3.13
**Pb 1ML**	90%	13	9.37	30%	53	1.71
	92%	14	10.78	48%	36	2.54
	102%	14	12.37	62%	53	2.89
**Pb 2ML**	97%	13	10.4	56%	56	3.18
	90%	14	11.88	94%	48	4.34
	97%	14	10.99	50%	39	2.57
**Pb 2ML***	99%	13	13.36	80%	43	3.55
	104%	13	12.09	50%	43	2.37
	92%	13	13.23	86%	43	5.09
**Cd ½ML**	88%	21	3.95	42%	53	2.22
	91%	21	4.07	44%	39	1.85
	72%	21	4.65	38%	43	2.07
**Cd 1ML**	98%	12	9.97	58%	46	3.45
	94%	12	11.1	74%	48	3.42
	88%	13	10.41	74%	41	3.81
**Cd 2ML**	98%	13	9.28	60%	50	3.36
	93%	13	10.61	74%	41	4.26
	84%	14	11.48	58%	46	2.33
**Cd 2ML***	106%	13	11.36	78%	43	3.06
	94%	13	15.11	60%	43	2.74
	96%	13	12.54	80%	43	3.96
